# Supplementary material for: Mass casualty incident preparedness and response: A desk review of the Code Orange Plan and Assessment of Healthcare Workers’ Knowledge, Attitudes, and Practices in a Lebanese Tertiary Government Hospital
Source: PLoS One. 2026 Apr 24;21(4):e0348176. doi: 10.1371/journal.pone.0348176 (PMC13108888; doi:10.1371/journal.pone.0348176)
Supplement: S1 Table — (DOCX) [file pone.0348176.s001.docx]

**Supplementary Table 1 Comparative Analysis of Code Orange Plan Against Key Guidelines**

| Themes | Key Areas | WHO Guidelines | CDC Guidelines | U.S. Department of Homeland Security's National Incident Management System (NIMS) | Association for Professionals in Infection Control and Epidemiology (APIC) | Alignment  of RHUH Code Orange | Deviation  of RHUH Code Orange |
| --- | --- | --- | --- | --- | --- | --- | --- |
| Preparedness | **Planning & Activation** | Contingency planning is an embedded part of the organizational structure of the incident command system but there is no separate structure for planning. | Presence of a collaborative and coordinated crisis plan. Plans, protocols, checklists,  and signs facilitate hospital management and minimize chaos during emergencies is essential. Additionally, it outlines the leadership structure of the plan, specifying who is responsible for managing and directing operations during an emergency. | Preparedness includes all actions needed to build and maintain the capability to manage various incidents. It involves an ongoing cycle of planning, training, equipping, exercising, evaluating, and making improvements. | It addresses foundational considerations for MCI that include disaster planning, disaster planning committees and administrative members, and some detailed actions regarding cooperating with external actors. | It mentions who is responsible for activating the plan. | There is no mention of the levels of alert or the activation stages of the plan. |
|  | **Surge Capacity** | It is defined as the ability of a health service to expand beyond normal capacity to meet increased demand for clinical care. It is important to address this in the MCI plan. | It includes surge capacity.  It also explains the patient distribution during MCI. It also maps the available resources. |  | This is explained more under the response about the hospital’s capacity to respond to patient influx. |  | Nothing is mentioned about procedures related to surge capacity. |
|  | **Continuity of essential services** | The availability of essential services needs to continue in parallel with activating a hospital emergency response plan. |  |  | Their part in relocating patients to ensure continuity of care | The Code Orange plan is more focused on the responsibility of staff rather than the procedures of services | Nothing is mentioned about the implementation of services. |
|  | **Evaluation and Drills** |  | It includes exercise and drills where Performance in drills and exercises should be methodically evaluated with input from other Hospitals, health care systems, and response agencies. | This falls under the preparedness section, which highlights that effective incident management starts with a range of preparedness activities, including thorough planning, training, and exercises. | It is highlighted under education and training about the importance of having mandatory disaster training programs. | There is a statement about applying drills every month | No details about how evaluation and drills will be applied. |
|  | **ER Evacuation Protocols** |  | It includes a response protocol for the Mass casualty event site. It also includes workflow during emergencies at the ER. |  | The plan should outline an organized discharge routine capable of managing large numbers of patients on short notice. |  | Nothing is mentioned about the evacuation protocol. |
| Incident Command System & Response | **Command and Control** | This refers to the structured management and coordination system that ensures an organized response to an emergency. | It explains the transition from reactive to initiative-taking where responsibilities are defined during an emergency. It includes an incident command system that defines responsibilities, prioritizes responses based on a predictable chain of management, and develops clear reporting channels and common nomenclature to facilitate communication. | It includes two levels of commands: incident command system and multiagency coordination systems. | The Hospital Disaster Control Command Center plan should include its designated location, an alternate site, a clear chain of command, and detailed standard operating procedures for coordination during emergencies. | Roles and responsibilities are defined for each personnel. There is a list of persons to be called. | There is no organized flow of actions. |
|  | **Triage Protocols** | Maintaining patient triage operations, based on a well-functioning mass-casualty triage protocol, is essential for the appropriate organization of patient care. | To provide care for the greatest number of patients, physicians may need to alter standards of triage and individual-level care to maximize the number of lives saved and minimize the consequences of  nonfatal injuries. |  | Triage is more defined under the reception of casualties and victims. | It is just mentioned who is going to perform triage | Triage protocols are not well-defined |
|  | **Surveillance** |  | It explains the necessity of having a casualty tracking system for registering and documenting purposes. | It is little mentioned under supporting technologies to maintain data. | The plan should establish a baseline of patient numbers in the Emergency Department, outpatient clinics, and direct admissions, categorized by clinical symptoms. | It is just mentioned who is going to collect the data | It is not well-defined |
|  | **Activation Procedures & Response** | This is embedded in the command system. |  | Activation is more defined under Incident Command. | The plan should clearly define the circumstances for activation and specify the position holder with the authority to activate or deactivate it. This also includes the hospital response plan and action. | It is mentioned who will activate it. | No detailed procedures for activation and alert systems in case of mass casualty incident |
| Communication | **Communication Channels** | It is necessary to ensure informed decision-making,  effective collaboration and cooperation, and public awareness and trust | It is mostly focused on external communication with local authorities and media. | It includes an integrated communication system for internal and external. | The plan should include provisions for handling overloads or failures of normal communication systems such as telephones, cellular phones, and pagers during disasters. It must specify alternative communication arrangements. This applies to internal and external. | An annex of the list of persons involved in “Code Orange” who must be called by telephone is attached to the plan. | - Lacks alternative arrangements for failed or overloaded communication systems. - The annex with “Code Orange” contacts does not detail breakdown handling. - Missing comprehensive strategy for external communication with authorities and media. |
| Management | **Safety & security** | Well-developed safety and security procedures are essential for the maintenance of hospital functions and for incident response operations during a disaster. | Hospitals should maintain control and security within their boundaries as law enforcement resources may be severely taxed. |  | This ensures the ability to control facility access through lockdown procedures, manage entry and exit points, regulate vehicular and pedestrian traffic, and facilitate clear communication with those outside during lockdown. | The roles of guard and security personnel are mentioned | No clear procedure for maintaining safety and security |
|  | **Human Resource** | Effective human resource management is essential to ensure adequate staff capacity and the  continuity of operations during any incident that increases the demand for human resources | It outlines the human resource needs based on whom to call and how to schedule changes. | It is mentioned that under emergency operations centers it should be staffed by personnel representing multiple functions. | The plan should define the roles and needs of key internal personnel. | The procedure is divided into roles of responsibilities of assigned personnel during MCI and there is an annex attached to the plan about the Code Orange team. |  |
|  | **Logistics and supply management** | Continuity of the hospital supply and delivery chain is often an underestimated challenge during a disaster, requiring attentive contingency planning and response. | It further emphasizes the importance of having redundant systems in place. Hospitals should develop contingency plans that include backup power sources, safeguarding communication and IT infrastructure, and well-defined evacuation and shelter-in-place procedures. | It includes supporting technologies that are under the logistics | The plan should detail the current availability of critical equipment. It must specify the facility’s stock levels of medical supplies. There should be procedures in case of a cut-off. |  | The plan lacks detailed procedures for critical actions such as equipment support, |
|  | **Resource Allocation** |  | It includes resource management, especially for medical supplies and blood banks. | Resource management involves the coordination and oversight of personnel, tools,  processes, and systems that provide incident managers with timely and appropriate resources during an incident | In response, the plan has mentioned about use of supplies during MCI. | There are attachments for annexes about the Code Orange file, a list of drugs in the pharmacy, and a Code Orange kit. | No Resource management is well explained and identified in the plan. |
|  | **Post-disaster recovery** | Post-disaster recovery planning should be performed at the onset of response activities.  Prompt implementation of recovery efforts can help mitigate a disaster’s long-term impact on  hospital operations. | It includes recovery to return to routine activities from emergency status quickly. | This is little addressed under the preparedness section, where recovery plans are mentioned, but there is no comprehensive section dedicated to it. | The plan should include a recovery phase following the MCI response to restore normal operations and return all services to their standard levels, including the replenishment of inventory supplies. |  | Nothing is mentioned about the post-disaster recovery and how the emergency will be closed. |
